# Community-Acquired *Staphylococcus argenteus* Sequence Type 2250 Bone and Joint Infection, France, 2017

**DOI:** 10.3201/eid2410.180727

**Published:** 2018-10

**Authors:** Josselin Rigaill, Florence Grattard, Sylvain Grange, Fabien Forest, Elie Haddad, Anne Carricajo, Anne Tristan, Frederic Laurent, Elisabeth Botelho-Nevers, Paul O. Verhoeven

**Affiliations:** University Hospital of Saint-Etienne, Saint-Etienne, France (J. Rigaill, F. Grattard, S. Grange, F. Forest, E. Haddad, A. Carricajo, E. Botelho-Nevers, P.O. Verhoeven);; Jean Monnet University, Saint-Etienne (J. Rigaill, F. Grattard, A. Carricajo, E. Botelho-Nevers, P.O. Verhoeven);; International Centre for Infectiology Research, Lyon, France (A. Tristan, F. Laurent);; French National Reference Centre for Staphylococci, Lyon (A.Tristan, F. Laurent)

**Keywords:** Staphylococcus argenteus, Staphylococcus aureus, bacteria, sequence type, ST2250, bone and joint infection, community-acquired infection, France

## Abstract

We report a rare case of *Staphylococcus argenteus* bone and joint infection in a 9-year-old boy in France. His finger arthritis was complicated by osteitis 5 weeks later, which resulted in a secondary intervention. This case indicates the virulence of *S. argenteus*, an emerging pathogen whose clinical effects are poorly described.

*Staphylococcus argenteus* (formerly *S. aureus* clonal complex 75) is an emerging species in the *S. aureus* complex (*1*). Several studies reported sporadic cases of *S. argenteus* infections mainly in Asia, Oceania, and the Pacific Islands (*2*) but rarely in Europe (*3*). We report the clinical characteristics of a community-acquired bone and joint infection with *S. argenteus* in a child living in France.

At the end of July 2017, a 9-year-old boy with no unusual medical history or previous local trauma was hospitalized because of acute signs of infection of the third finger on his right hand. He was first seen in a local hospital and given an initial diagnosis of cellulitis (arthritis). Two days later, he was admitted to the emergency pediatric ward of a tertiary care hospital where a surgical joint exploration was performed and confirmed the diagnosis of arthritis associated with an abscess of the extensor tendon sheath (Table).

**Table:** Clinical characteristics and timeline for patient with community-acquired *Staphylococcus argenteus* sequence type 2250 bone and joint infection, France, 2017*

Characteristic	Jul 30	Aug 2	Aug 8	Sep 5	Nov 2
Hospital	Local	Tertiary care	Tertiary care	Tertiary care	Tertiary care
Clinical features	Pain in third finger of right hand	Fever (temperature 38.6°C); pain and functional impotence in flexion of finger	Poor tolerance of antimicrobial drugs	Fever (temperature 38.4°C); pain in finger	Stiffness in finger; no pain
Signs at physical examination	Inflammatory edema of finger; no inoculation lesion	Phlegmon of finger: inflammatory skin; edema on second phalanx	ND	Misalignment of second phalanx	No signs of infection
Laboratory findings					
Leukocytes, ×1 0^9 ^cells/L†	20	7.2	ND	11.6	7.2
C-reactive protein, mg/L‡	58	17.7	ND	ND	0.3
Microbiological	Culture of infection site not performed (no pus); blood culture not performed	Surgical samples: neutrophils and gram-positive cocci (identified as *S. argenteus*); blood cultures sterile	ND	Surgical samples: few neutrophils and negative gram staining results; culture remained sterile after 10 d; negative 16S rDNA PCR result; blood cultures not performed	ND
Radiologic findings	Not performed	Radiograph of hand: no signs of osteitis	ND	MRI of hand: osteitis	ND
Histologic findings			ND	Chronic osteitis	ND
Diagnosis considered	Cellulitis of finger	Arthritis of second phalanx; abscess of extensor tendon sheath	ND		ND
Treatment					
Antimicrobial drugs	AMX (1,000 mg/d) and CLA (125 mg/d)	CFZ (2,200 mg/d) and GEN (400 mg/d) for 2 d; AMX (2,000 mg/d), CLA (250 mg/d), RIF (600 mg/d) for 6 wk	Stop AMX and CLA; FUS (1.5 g/d) and RIF (600 mg/d) for 5 wk	Sep 8: CLI (900 mg/d) and OFX (400 mg/d) for 6 wk	ND
Surgery		Surgical joint lavage and débridement for massive purulent abscess that reached the extensor tendon and joint capsule of second phalanx; no articular cartilage lesion	ND	Surgical lavage and realignment of phalanxes with implantation of external fixator on Sep 8	ND
Outcome	Discharged	Improvement at discharge on Aug 4; patient seen on Aug 6, 8, 10, and 12	Good outcome	Discharged on Sep 11; patient seen on Sep 13, 15, and 22, and Oct 6; external fixator removed on Oct 6	Good outcome and functional rehabilitation; patient seen on Dec 27 and had similar findings

*AMX, amoxicillin; CFZ, cefazolin; CLA, clavulanic acid; CLI, clindamycin; FUS, fusidic acid; GEN, gentamicin; MRI, magnetic resonance imaging; ND, not determined; OFX, ofloxacin; RIF, rifampin. †Reference range 4.5–13.5 × 10^9 ^cells/L. ‡Reference value <5 mg/L.

Surgical microbiological samples cultured on blood agar plates (aerobic conditions at 37°C for 24 h) grew a strain that was identified by matrix-assisted laser desorption/ionization time-of-flight mass spectrometry (Microflex LT, Bruker, France) as having log scores ranging from 1.39 to 1.87 (corresponding to *S. aureus*, *S. simiae*, or *S. epidermidis*). These scores were lower than those required for reliable identification of species (2.3) or genus (2.1) levels. Antimicrobial drug susceptibility testing (Vitek2; bioMérieux, Marcy l’Etoile, France) identified resistance to penicillin G.

Because there was no initial reliable identification of this strain, we performed molecular tests. The strain was negative for *nuc*, *lukS*-PV, *mec*A, *mec*C, *tst*-1, and *spa* genes. The V3 region sequence of the gene coding for 16 SrRNA showed 100% identity with that for *S. aureus*. Microarray analysis (*S. aureus* Genotyping Kit 2.0; Alere Technologies GmbH, Jena, Germany) assigned this strain to the clonal complex 2250/2277 of one of the main clusters of *S. argenteus* (*2*,*4*). The patient was discharged and received an oral antimicrobial regimen for 6 weeks; healing was closely monitored (Table).

Five weeks later, the patient was hospitalized because of recurrent signs of infection (Table). Magnetic resonance imaging of the right hand showed osteitis (Technical Appendix, panel A). A second surgical procedure was performed (Table). Cultures of surgical samples remained sterile after 10 days and a 16S rDNA PCR result was negative. Histologic analysis showed chronic osteitis (Technical Appendix Figure, panel B). An oral drug regimen, including clindamycin and ofloxacin for 6 weeks, was prescribed. Long-term outcome was good (Table), despite persistence of stiffness in the finger.

This rare case of osteomyelitis caused by *S. argenteus* highlights the difficulties in correctly identifying this species. As with *S. schweitzeri*, *S. argenteus* is an emergent species that has been described as part of the *S. aureus* species complex (*5*). *S. argenteus* was first described in 2002 as a CC75/sequence type T1223 clone of *S. aureus* in Aboriginal communities in Australia (*6*). This species was named *S. argenteus* in 2011 because of its lack of staphyloxanthin production (*1*). Most studies reported prevalence rates for *S. argenteus* among strain collections of 0.16%–18.6% according to geographic distribution, with a clear predominance in Asia and the West Pacific region (*1**,**5*–*7*) and a rare description in Europe (*3*). 

In our case, no epidemiologic link to Asia or the West Pacific region was observed. The clinical spectrum of infections with *S. argenteus* remains poorly described, but varies from asymptomatic nasal carriage (*8*) to community-acquired infections, including skin and soft tissue infections (*1*,*6*), bacteremia (*1**,**9*), and foodborne illness (*8*). Apart from 2 cases of bacteremia reported by Dupieux et al. (*9*), clinical data for *S. argenteus* infections have been poorly detailed (Technical Appendix Figure). To the best of our knowledge, only 1 case of osteomyelitis has been reported (*7*).

Previous cases and the case we report indicate that *S. argenteus* could be responsible for invasive infections that are difficult to manage. *S. argenteus* was initially considered to be less virulent than *S. aureus* on the basis of a study in Australia, which reported that this species was associated mainly with skin and soft tissue infections and rarely with bacteremia (3/220 cases) (*1*). However, a comparative study of 311 cases of *S. argenteus* and *S. aureus* sepsis in Thailand showed a similar outcome after 28 days (*10*). Moreover, virulence factors, such as Panton-Valentine leucocidin and enterotoxins, have been described in *S. argenteus* isolates (*4*,*8*–*10*). In contrast to reports from Aboriginal communities in Australia (*6*) and remote populations in the West Pacific region (Technical Appendix Table), resistance to methicillin was not detected in strains from the case-patients in this study.

In contrast to *S. aureus*, the effect of carriage of *S. argenteus* has not been studied. For our case-patient, screening for *S. argenteus* nasal carriage was not performed. However, a recent study of foodborne illness outbreaks reported the ability of this bacterium to spread in the environment and colonize food handlers (*8*).

*S. argenteus* is an emerging species for which its clinical spectrum remains poorly described. Further studies are needed to better address the global prevalence and clinical role of this bacterium, including its potential effects in chronic human infections.

Technical Appendix Additional information on community-acquired *Staphylococcus argenteus* sequence type 2250 bone and joint infection, France 2017.
