# Supplementary material for: Community-Acquired Staphylococcus argenteus Sequence Type 2250 Bone and Joint Infection, France, 2017
Source: Emerg Infect Dis. 2018 Oct;24(10):1958–61. doi: 10.3201/eid2410.180727 (PMC6154148; doi:10.3201/eid2410.180727)
Supplement: Technical Appendix — Additional information on community-acquired Staphylococcus argenteus sequence type 2250 bone and joint infection, France 2017. [file 18-0727-Techapp-s1.pdf]

Article DOI: https://doi.org/10.3201/eid2410.180727

Community-Acquired *Staphylococcus argenteus* Sequence Type 2250 Bone and Joint Infection, France 2017

Technical Appendix

Technical Appendix Table. Characteristics of reported cases of infection with *Staphylococcus argenteus**

Reference	No. cases	Patients	Signs/symptoms	Diagnostic method	Bacterial strain characteristics	Treatment	Prognosis
Holt et al., 2011 (*1*)	1	Woman from Australia	Necrotizing fasciitis and bacteremia	WGS	Resistant to methicillin	Not described	Not reported
Dupieux et al., 2015 (*2*)	2	25-year-old woman	Pulmonary and bacteremia	Microarray, MLST	Susceptible to methicillin, positive for PVL	Amoxicillin/clavulanic acid, roxithromycin, ceftriaxone, spiramycin, oxacillin, clindamycin, linezolid	Recovered
		18-month-old child	Knee arthritis, bacteremia, and multiple pulmonary abscesses			Amoxicillin/clavulanic acid, gentamicin, clindamycin, linezolid, oxacillin, rifampin, polyvalent immunoglobulin	
Thaipadungpanit et al., 2015 (*3*)	10	270 in Thailand	2 with bacteremia; 7 with SST; 1 with osteomyelitis (2 healthcare-associated infections	SNP	All resistant to methicillin; none positive for PVL	Not described	Same prognosis as *S. aureus* infections for death and illness
Chantratita et al., 2016 (*4*)	58	311 in Thailand	23 with bacteremia	PFGE, MLST	No strain resistant to methicillin; 15 strains positive for PVL	Not described	Same death rate as for *S. aureus* infections
Suzuki et al., 2017 (*5*)	2	Intoxicated 3 h after eating lunch	Food poisoning	WGS	Both strains produced SEB; no SCCmec elements	Not described	Not described
Wakabayashi et al., 2018 (*6*)	4 in 2014; 13 in 201	3 patients and 1 food handler in 2014; 12 patients and 1 food handler in 2015	Food poisoning	PFGE, MLST	All strains positive for SEB	Not described	Not described

Reference	No. cases	Patients	Signs/symptoms	Diagnostic method	Bacterial strain characteristics	Treatment	Prognosis
This study	1	9-year-old boy in France	Finger arthritis and osteomyelitis	Microarray	Susceptible to methicillin	Amoxicillin/clavulanic acid, ceftazolin, gentamicin, rifampin, fusidic acid, clindamycin, ofloxacin	Recovered

*MLST, multilocus sequence typing; PFGE, pulsed-field gel electrophoresis; PVL, Panton-Valentine leukocidin; SCCmec, staphylococcal cassette chromosome mec; SEB, staphylococcal enterotoxin B; SNP, single-nucleotide polymorphism; SSTI, skin and soft tissue infection; WGS, whole-genome sequencing.

References

1. Holt DC, Holden MT, Tong SY, Castillo-Ramirez S, Clarke L, Quail MA, et al. A very early-branching *Staphylococcus aureus* lineage lacking the carotenoid pigment staphyloxanthin. Genome Biol Evol. 2011;3:881–95. PubMed http://dx.doi.org/10.1093/gbe/evr078

2. Dupieux C, Blondé R, Bouchiat C, Meugnier H, Bes M, Laurent S, et al. Community-acquired infections due to *Staphylococcus argenteus* lineage isolates harbouring the Panton-Valentine leucocidin, France, 2014. Euro Surveill. 2015;20:21154. PubMed http://dx.doi.org/10.2807/1560-7917.ES2015.20.23.21154

3. Thaipadungpanit J, Amornchai P, Nickerson EK, Wongsuvan G, Wuthiekanun V, Limmathurotsakul D, et al. Clinical and molecular epidemiology of *Staphylococcus argenteus* infections in Thailand. J Clin Microbiol. 2015;53:1005–8. PubMed http://dx.doi.org/10.1128/JCM.03049-14

4. Chantratita N, Wikraiphat C, Tandhavanant S, Wongsuvan G, Ariyaprasert P, Suntornsut P, et al. Comparison of community-onset *Staphylococcus argenteus* and *Staphylococcus aureus* sepsis in Thailand: a prospective multicentre observational study. Clin Microbiol Infect. 2016;22:458.e11–9. PubMed http://dx.doi.org/10.1016/j.cmi.2016.01.008

5. Suzuki Y, Kubota H, Ono HK, Kobayashi M, Murauchi K, Kato R, et al. Food poisoning outbreak in Tokyo, Japan caused by *Staphylococcus argenteus.* Int J Food Microbiol. 2017;262:31–7. PubMed http://dx.doi.org/10.1016/j.ijfoodmicro.2017.09.005

6. Wakabayashi Y, Umeda K, Yonogi S, Nakamura H, Yamamoto K, Kumeda Y, et al. Staphylococcal food poisoning caused by *Staphylococcus argenteus* harboring staphylococcal enterotoxin genes. Int J Food Microbiol. 2018;265:23–9. PubMed http://dx.doi.org/10.1016/j.ijfoodmicro.2017.10.022

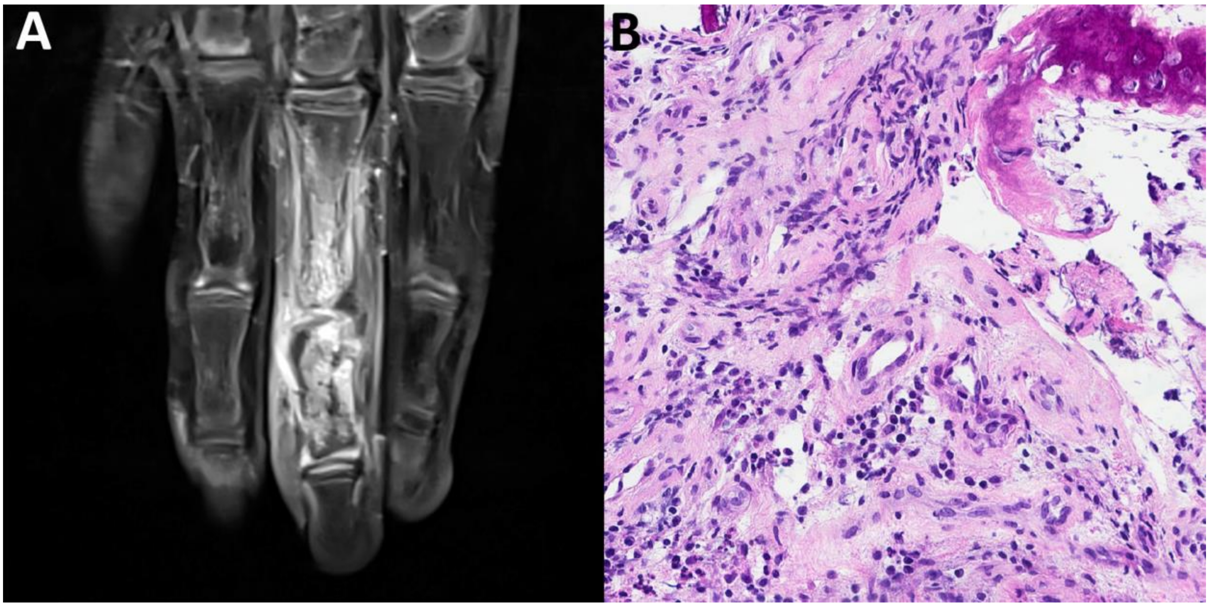

Technical Appendix Figure. Community-acquired *Staphylococcus argenteus* sequence type 2250 bone and joint infection in a 9-year-old boy, France 2017. A) Fat-saturated, T1-weighted, magnetic resonance image after gadolinium injection in coronal plane of the right hand, showing evidence of osteitis at the base of middle phalanx up to cartilaginous growth plate of third finger with local necrosis at the time of infection. B) Infected bone tissue showing infiltration with numerous plasmocytes and lymphocytes on the left at the time of infection (hematoxylin and eosin stained, original magnification ×100).
